# Morphologic and Pharmacological Investigations in the Epicatechin Gastroprotective Effect

**DOI:** 10.1155/2012/708156

**Published:** 2012-05-14

**Authors:** A. L. Rozza, C. A. Hiruma-Lima, A. Tanimoto, C. H. Pellizzon

**Affiliations:** ^1^Morphology Department, Biosciences Institute, UNESP-University Estadual Paulista, P.O. Box 510, 18618-970 Botucatu, SP, Brazil; ^2^Physiology Department, Biosciences Institute, UNESP-University Estadual Paulista, 18618-970 Botucatu, SP, Brazil

## Abstract

Previous studies of the gastroprotective activity of plants have highlighted the importance of the polyphenolic compound epicatechin (EC) in the treatment of gastric ulcers. This paper aimed to evaluate and characterize the gastroprotective mechanism of action of EC using male rats. The gastroprotective action of EC was analyzed in gastric ulcers induced by ethanol or indomethacin. The involvement of sulfhydryl (SH) groups, K^+^_ATP_ channels, *α*_2_ adrenoceptors, gastric antisecretory activity, and the amount of mucus in the development of gastric ulcers were investigated. The lowest effective dose of EC providing gastroprotective effects was 50 mg/kg in the ethanol-induced gastric ulcers and 25 mg/kg in the indomethacin-induced gastric ulcers. The gastroprotection seen upon treatment with EC was significantly decreased in rats pretreated with a SH compound reagent or an *α*_2_-receptor antagonist, but not with a K^+^_ATP_ channel blocker. Furthermore, oral treatment with EC increased mucus production and decreased H^+^ secretion. Immunohistochemistry demonstrated the involvement of superoxide dismutase (SOD), nitric oxide (NO), and heat shock protein-70 (HSP-70) in the gastroprotection. These results demonstrate that EC provides gastroprotection through reinforcement of the mucus barrier and neutralization of gastric juice and this protection occurs through the involvement of SH compounds, *α*_2_-adrenoceptors, NO, SOD, and HSP-70.

## 1. Introduction

Gastric ulcers affect thousands of people worldwide and are considered a global health problem. The pathophysiology of gastric ulcers is related to the disequilibrium between harmful and protective factors in the gastric mucosa. Agents that may initiate the development of an ulcer include the following: acid and pepsin secretion, *Helicobacter pylori* infection, poor diet, alcohol ingestion, and the use of nonsteroidal anti-inflammatory drugs (NSAIDs). Protective factors include the following: an intact mucosal barrier, adequate mucus secretion and blood flow, cellular regeneration, prostaglandin secretion, and epidermal growth factor release [1, 2].

The current treatment options for patients suffering from gastric ulcers include antacids, sucralfate, prostaglandins, muscarinic antagonists, histamine-2 receptor antagonists, and proton pump inhibitors. However, the long-term use of these drugs may cause side effects such as hypersensitivity, arrhythmia, impotence, gynecomastia, and hematopoietic disturbances [3]. Additionally, their use does not necessarily prevent the recurrence of the disease.

*H. pylori* infection has been implicated in the pathogenesis of active and chronic gastritis, peptic ulcer, and gastric carcinoma [4]. The triple therapy, based on a proton pump inhibitor combined with clarithromycin and amoxicillin and/or metronidazole, has been the established first-line therapy over the past years to eradicate *H. pylori* infections [5–7]. Standard triple therapy started from eradication rates of more than 90%; however, until now it has experienced a steady decline, decreasing to 70–80% [8]. There are several reasons for the loss of eradication efficacy, but the most important is the increasing rate of *H. pylori* resistance to antibiotics [9].

This circumstance evidences the need for new research to discover substances that would effectively heal gastric ulcers with fewer side effects. The medicinal plants are attractive sources of new biomolecules, particularly in the developing world where infectious diseases are endemic and modern health facilities are not always accessible [10]. Although modern medicine may be available in many developing countries, natural medicines have maintained popularity for historical and cultural reasons. Concurrently, many people in developed countries have begun to turn to complementary or alternative therapies, including medicinal herbs [11, 12].

Polyphenols, which include flavonoids and tannins, are a group of phytochemicals that have been intensely researched and are known to exert beneficial effects on health [13], including experimental gastroprotective action.

**Figure 1:**
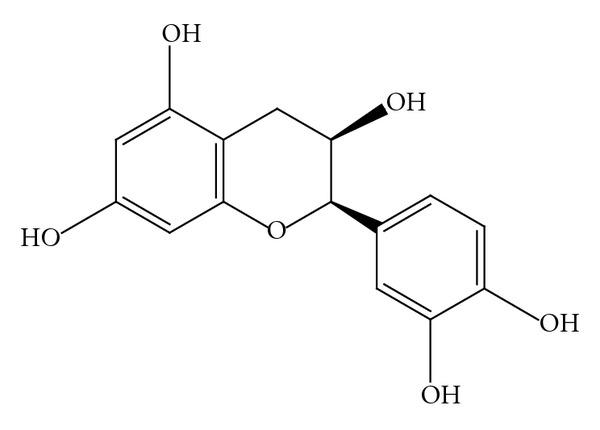
Chemical structure of epicatechin.

Epicatechin (EC, Figure 1), an isomer of catechin, is a polyphenolic compound present in several plant species. Previous studies have shown the gastroprotective activity of *Mouriri pusa* [14] in which the EC was the main compound of the extract. Based upon this research, we sought to characterize the mechanism of action of EC in gastroprotection.

## 2. Material and Methods

### 2.1. Epicatechin: Determination of Doses and Vehicle Used

(−)-Epicatechin (catalog number 855235) was purchased from Sigma Chemical Co. (St. Louis, MO, USA). In ethanol and indomethacin-induced gastric ulcers, the following doses were tested: 25, 50, and 75 mg/kg. The lowest effective dose was used for all subsequent experiments.

Saline +10% absolute ethanol was used as a vehicle, as EC is insoluble in 0.9% saline. To avoid differences between groups, all drug compounds were solubilized in this vehicle, including the positive controls.

### 2.2. Animals

Male Wistar rats (200–250 g) from the Central Animal House of UNESP were fed a certified diet with free access to tap water under standard light-dark cycles (12 h dark-12 h light), humidity (60 ± 1%), and temperature (21 ± 2°C). All rats were fasted prior to each experiment as treatments were orally administered. Additionally, the rats were housed in cages with raised floors of wide mesh to prevent coprophagy. All experimental protocols followed the recommendations of the Canadian Council on Animal Care and were approved by the UNESP Institutional Animal Care and Use Committee.

### 2.3. Experimental Assays

#### 2.3.1. Ethanol-Induced Gastric Ulcers

Male Wistar rats that had been fasted for 24 h were distributed into six groups (*n* = 7). Animals were then orally dosed with vehicle (10 mL/kg), carbenoxolone (100 mg/kg), or EC (25, 50, or 75 mg/kg). The sixth group (sham) did not receive either drug or vehicle. After 1 hour, the animals received an oral dose of 1 mL of absolute ethanol. One hour after ethanol treatment, the rats were sacrificed and their stomachs were removed. The stomachs were then opened along the greater curvature and washed. The flattened stomach samples were scanned and the ulcer area (mm^2^) was measured using AVSoft BioView software. A small fragment of each stomach was collected for glutathione measurement. Stomach samples were collected for histological slide preparation and stained with hematoxylin and eosin (HE) to analyze the morphological and histological characteristics, or the slides were used for immunohistochemical analyses. A microscopic score [15] was determined for the following parameters: epithelial desquamation, hemorrhage, glandular damage, and eosinophilic infiltration, using a scale ranging from 0 to 3 (0: none, 1: mild, 2: moderate, and 3: severe) for each criterion. The highest possible score was 12.

**Immunohistochemical Analysis**

Six slides were used for each antibody. Each slide was deparaffinized, rehydrated, and immunostained by the ABC method. Nonspecific reactions were blocked with H_2_O_2_ and goat serum prior to incubation with a specific antibody. After rinsing in phosphate-buffered saline (PBS, 0.01 Mol/L, pH 7.4), the sections were incubated in secondary antibody (ABC kit, Easypath Erviegas). The sections were then washed in PBS buffer, the ABC complex was applied, and the reaction was carried out in a DAB solution (3,3′-diaminobenzidine-tetrahydrochloride) containing 0.01% H_2_O_2  _in PBS buffer. After immunostaining, the sections were lightly counterstained with hematoxylin and the immunoreactive cells were observed under a Leica microscope using Leica QWin Software (Leica, UK). In the control reaction, the slides were processed without the primary antibody or in the absence of all antibodies. The slides were stained with antibodies for heat-shock protein 70 (HSP-70), superoxide dismutase (SOD), and nitric oxide (NO) (Santa Cruz Biotechnology). The marked area (*μ*m^2^) in each slide was measured using AVSoft BioView software.

**Determination of Total Glutathione Levels**

The total glutathione content in the stomach was quantified using the recycling assay [16]. Stomach samples containing ethanol-induced gastric ulcers were thawed and minced, diluted 1 : 20 (w/v) in ice-cold 5% (w/v) trichloroacetic acid, and homogenized. The homogenates were centrifuged at 7000 g for 15 minutes at 4°C. The resulting supernatant was used to quantify total glutathione content by reaction with DTNB (5,5′-ditiobis-2-nitrobenzoic acid). Total glutathione was quantified by measuring the absorbance at 412 nm. The results were expressed as nmol total glutathione/g tissue.

#### 2.3.2. Involvement of Sulfhydryl (SH) Compounds in Gastroprotection

Rats were distributed into four groups (*n* = 7). Two groups of rats were intraperitoneally treated with NEM (7 mg/kg), an SH compound blocker. The other two groups were treated with vehicle (10 mL/kg). One hour later, either vehicle or EC (50 mg/kg) was orally administered to two groups each. The ulcers were induced following the ethanol-induced gastric ulcer model and the ulcer area (mm^2^) was determined with the aid of AVSoft BioView software.

#### 2.3.3. Involvement of *K* ^+^_*ATP*_ Channels or Presynaptic *α*_2_-Receptors in Gastroprotection

Rats were distributed into six groups (*n* = 7). Two groups of rats were subjected to intraperitoneal treatment with the following drugs: the K^+^_ATP_ channel blocker glibenclamide (3 mg/kg), the *α*_2_-receptor antagonist yohimbine (3 mg/kg), or vehicle (10 mL/kg). One hour later, either vehicle or EC (50 mg/kg) was orally administered to two groups each. The ulcers were induced following the ethanol-induced gastric ulcer model and the ulcer area (mm^2^) was determined with the aid of AVSoft BioView software.

#### 2.3.4. NSAID- (Indomethacin-) Induced Gastric Ulcers

The rats were distributed into five groups (*n* = 7). Vehicle (10 mL/kg), cimetidine (100 mg/kg), or EC (25, 50, or 75 mg/kg) was orally administered 30 minutes prior to the induction of gastric lesions by oral administration of the ulcerogenic agent indomethacin (100 mg/kg). The animals were sacrificed 5 h after treatment with indomethacin [17]. The stomachs were removed, opened along the greater curvature, and then scanned. The ulcer area (mm^2^) was determined using AVSoft BioView software.

#### 2.3.5. Evaluation of Gastric Juice Parameters

Male rats were randomly divided into 6 groups (*n* = 7). Thirty minutes after oral treatment or immediately after the intraduodenal administration of a single dose of vehicle (10 mL/kg), cimetidine (100 mg/kg), or EC (50 mg/kg), the rats were subjected to pyloric ligation [18]. Four hours later, the animals were sacrificed, the abdomen opened, and another ligature was placed around the esophagus, close to the diaphragm. The stomach was removed and its contents were drained into a graduated centrifuge tube, which was centrifuged at 2000 g for 15 minutes. The total acid content of the gastric secretions was determined by titration to pH 7.0 with 0.01 N NaOH using a digital burette (E.M., Hirschmann Technicolor, Germany). The total concentration of acid was expressed as mEq/mL/4 h.

#### 2.3.6. Determination of Mucus Adherence to the Gastric Wall

After 24 h of fasting, anesthetized rats (*n* = 7) were subjected to longitudinal incisions slightly below the xiphoid apophysis to place a pyloric ligature. Oral administration of vehicle, carbenoxolone (200 mg/kg), or EC (50 mg/kg) was performed 1 h before the ligature. After 4 h, the animals were sacrificed and the glandular portion of the stomach was weighed and immersed in Alcian Blue solution for the mucus quantification procedure. The absorbance was measured in a spectrophotometer at a wavelength of 598 nm, and the results were expressed as *μ*g Alcian Blue/g tissue [19].

### 2.4. Statistical Analysis

Parametric data were analyzed using a one-way analysis of variance (ANOVA) followed by Dunnett's test or Tukey's test and compared to the vehicle group. The results were presented as the mean ± standard error of the mean (SEM). Nonparametric data (histology scoring) were analyzed by the Kruskal-Wallis (nonparametric ANOVA) test followed by a Dunn multiple comparison test. The results were presented as the median (range). All analyses were performed using GraphPad InStat software. A value of *P* < 0.05 was considered significant.

## 3. Results

### 3.1. Ethanol-Induced Gastric Ulcers

#### 3.1.1. Gastric Ulcer Area

**Figure 2:**
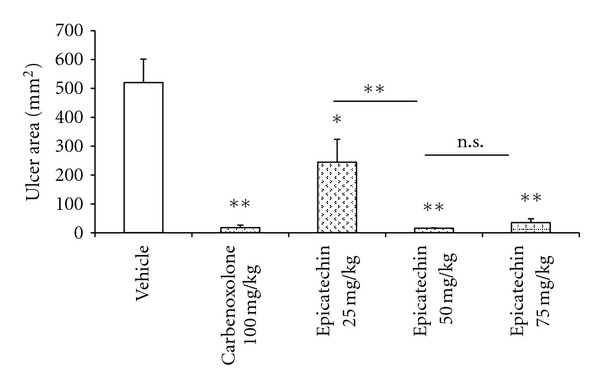
Gastric ulcer area (mm^2^) of rat stomachs (*n* = 7) with ethanol-induced gastric ulcers after treatment with vehicle, carbenoxolone (100 mg/kg), or epicatechin (25, 50 or 75 mg/kg). The results are reported as the mean ± SEM, analyzed by ANOVA followed by Dunnett's test, *P < 0.05 and **P < 0.01. ns: not significant.

The vehicle group presented several hemorrhagic bands, with an average ulcer area of 520.41 ± 81.46 mm^2^. The three doses of EC (25, 50, and 75 mg/kg) tested exhibited gastroprotective effects (*P* < 0.05 for the lowest dose and *P* < 0.01 for 50 and 75 mg/kg); however, the 50 mg/kg dose exhibited the greatest gastroprotective effect (94.83% gastroprotection). According to Tukey's test, there was no significant difference between the ulcer areas of the groups treated with 50 mg/kg or 75 mg/kg; therefore, the dose of 50 mg/kg was used for all subsequent experiments. The ulcer areas (mm^2^) are represented in Figure 2.

#### 3.1.2. Microscopic Score

**Figure 3:**
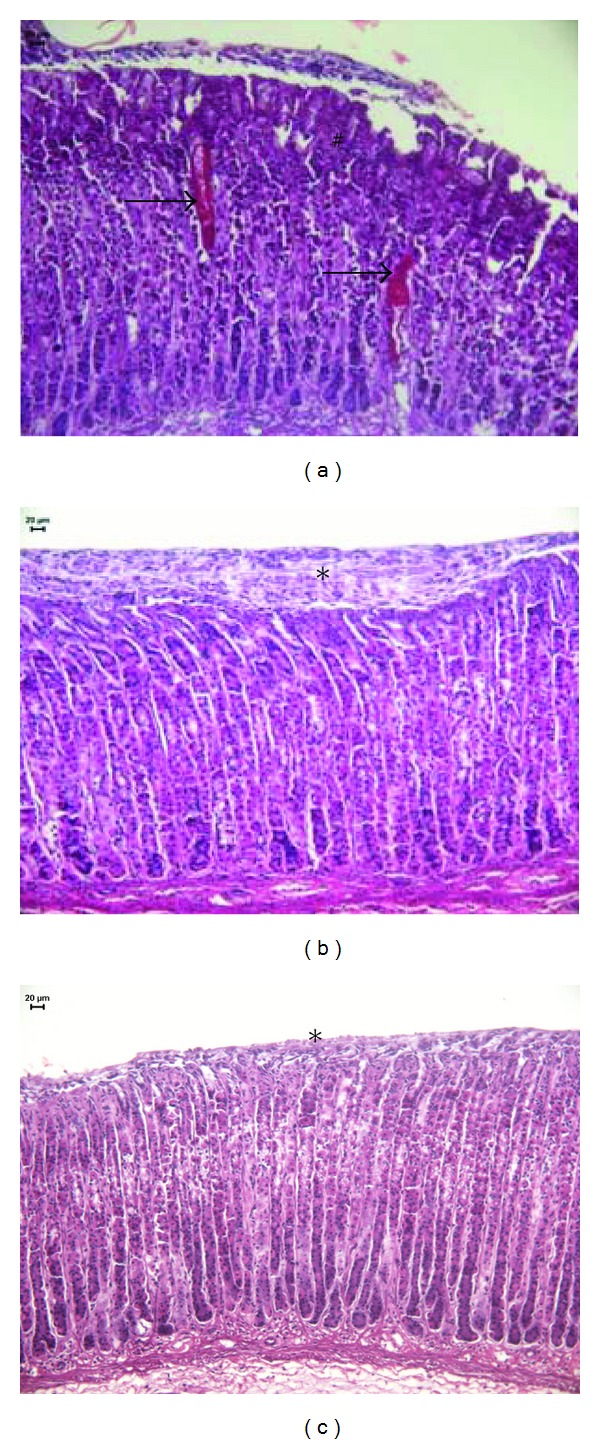
Photomicrography of rat stomachs with ethanol-induced gastric ulcers after treatment with (a) vehicle, (b) carbenoxolone (100 mg/kg), or (c) epicatechin (50 mg/kg). HE staining. *Indicates epithelial desquamation, ^#^indicates glandular damage, and the arrow indicates a hemorrhage arrows indicate hemorrhage.

There was no microscopic evidence of eosinophilic infiltration and hemorrhage, but there was mild desquamation and glandular damage in the group treated with 50 mg/kg EC. The HE staining of the ulcers is displayed in Figure 3 and the microscopic score is shown in Table 1.

#### 3.1.3. Immunohistochemistry

For HSP-70, SOD, and NO, the immunoreactive areas in the 50 mg/kg EC-treated group were statistically larger (*P* < 0.01) than those in the control group. These data indicate their involvement in the gastroprotective effect of treatment with EC (Table 1).

#### 3.1.4. Determination of Total Glutathione Levels

**Figure 4:**
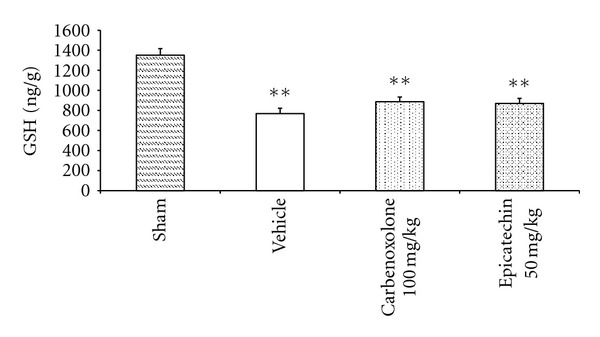
Glutathione levels (nmol total glutathione/g tissue) of rat stomachs (*n* = 7) with ethanol-induced gastric ulcers after treatment with vehicle, carbenoxolone (100 mg/kg), or epicatechin (50 mg/kg). The results are reported as the mean ± SEM, analyzed by ANOVA followed by Dunnett's test, compared to sham group, *P* < 0.01.

The treatment with EC (50 mg/kg) maintained the total glutathione level near that of the vehicle and carbenoxolone groups after the total glutathione-depleting ethanol administration (Figure 4). However, the total glutathione level in EC-treated animals was lower than that in the sham-treated group.

### 3.2. Involvement of NP-SH Compounds, K^+^_ATP_ Channels, or Presynaptic *α*_2_-Receptors in Gastroprotection

In rats pre-treated with NEM (an SH compound reagent), the gastroprotective effect of EC (50 mg/kg) was reversed (Table 2), indicating the involvement of SH compounds in gastroprotection. Rats pretreated with yohimbine (an *α*_2_-receptor antagonist) also exhibited a reversal in gastroprotection upon treatment with EC (Table 3), indicating the involvement of *α*_2_-receptors in the protective mechanism of action of EC (50 mg/kg). However, in rats pretreated with glibenclamide (a K^+^_ATP_ channel blocker), the gastroprotective effect of EC (50 mg/kg) was maintained (84.14%), indicating that there is no involvement of the K^+^_ATP_ channels in the gastroprotective effects of EC (Table 3).

### 3.3. Indomethacin-Induced Gastric Ulcers

**Figure 5:**
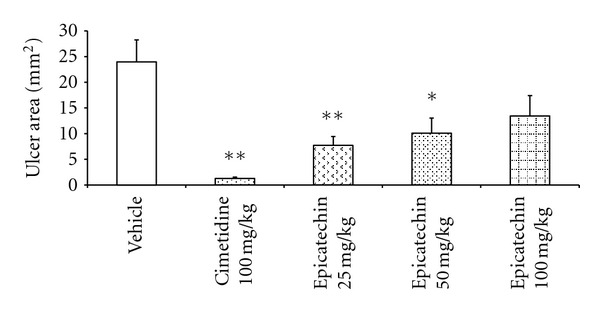
Gastric ulcer area (mm^2^) in rat stomachs (*n* = 7) with indomethacin-induced gastric ulcers after treatment with vehicle, carbenoxolone (100 mg/kg), or epicatechin (25, 50 or 75 mg/kg). The results are reported as the mean ± SEM, analyzed by ANOVA followed by Dunnett's test, compared to vehicle group, *P < 0.05 and **P < 0.01.

The vehicle-treated group had a large quantity of small petechiae in the stomach and a mean ulcer area of 23.97 ± 4.29 mm^2^. In this model, the most effective dose of EC was 25 mg/kg, which had a 67.83% gastroprotective effect (ulcer area 7.71 ± 1.74 mm^2^, *P* < 0.01). A dose of 50 mg/kg offered 57.90% gastroprotection (ulcer area 10.09 ± 2.93, *P* < 0.05) and the highest dose (75 mg/kg) was not gastroprotective (43.87%) (Figure 5).

### 3.4. Evaluation of the Gastric Juice Parameters

A comparison of the gastric juice parameters of the rats that were treated with EC (50 mg/kg) administered by oral or intraduodenal routes demonstrated that the oral treatment was able to diminish the H^+^ concentration in the gastric juice without modifying its volume. In comparison, the intraduodenal administration was not able to decrease the H^+^ concentration, but it did diminish the volume of the gastric juice (Table 4).

### 3.5. Determination of Mucus Adherence to the Gastric Wall

**Figure 6:**
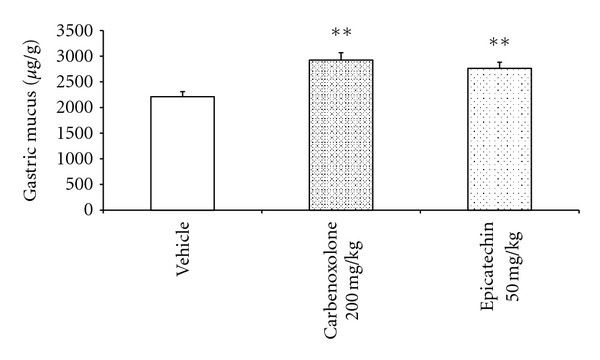
Quantification of adherent mucus (*μ*g/g of tissue) in the gastric mucosa of rats treated with vehicle, carbenoxolone (200 mg/kg), or epicatechin (50 mg/kg), analyzed by ANOVA followed by Dunnett's test, *P* < 0.01.

There was a significant increase in the amount of gastric mucus adhering to the stomach wall in the EC-treated (50 mg/kg) group (2763.07 ± 117.7 *μ*g/g, *P* < 0.01) versus the vehicle-treated group (2211.73 ± 98.04 *μ*g/g) (Figure 6).

## 4. Discussion

Commonly used therapies for the treatment of gastric ulcers often fail to completely heal the ulcers and additionally present with many side effects. Furthermore, patients are searching for new and natural methods of healing their diseases. Therefore, there has been an increased interest in the search for natural products to treat gastric ulcers. The goal of this work was to characterize the gastroprotective mechanism of action of epicatechin (EC), which is a polyphenolic compound found in several plant species.

Motawi et al. [20] previously demonstrated that an elevation in gastric acid secretion, neutrophil infiltration, and alterations in NO production and oxidative stress are mechanisms that contribute to indomethacin-induced ulceration. Indomethacin also induces gastric ulcer formation through the inhibition of PGE_2 _ synthesis, resulting in a decline in mucus production and subsequent hemorrhagic ulcers [21]. In the two lowest doses tested in this study, EC was able to alleviate the effects of indomethacin and prevent gastric ulcer formation. The effect of EC seen in this study was not dose-dependent, which is consistent with previously published studies on polyphenolic compounds [22–25]. This is most likely due to the antioxidant effect of lower doses, while oxidants may be produced at higher doses [26].

Orally administered ethanol causes injury to the gastric mucosa by decreasing local blood flow [27]. It also decreases the levels of total glutathione and SH, which are important gastroprotective factors [28], and depletes the gastric mucus [29] resulting in gastric lesions. In the ethanol-induced gastric ulcer assay, the EC protective effect at the three doses tested suggests a gastroprotective mechanism that acts directly in the gastric mucosal cells. The intermediate dose (50 mg/kg) was chosen for subsequent assays because its gastroprotective effect was significantly higher than the effect of the lower dose (25 mg/kg) and was not different from the highest dose (100 mg/kg).

The mechanisms that could be responsible for the gastroprotective effects were investigated as follows. Endogenous SH compounds are key agents in mucosal protection against ethanol-induced gastric injury [30]. These SH compounds provide protective effects through binding free radicals formed by ethanol treatment and by controlling the production of mucus [31]. Our results evidence the importance of these compounds in the gastroprotective mechanism of EC. Moreover, SH compounds are also involved in the maintenance of the mucus disulfide bridges that, if damaged, render the mucus more soluble, resulting in a gastric mucosa that is more susceptible to injuries caused by harmful agents [32]. The involvement of this pathway in gastroprotection may also explain why the treatment with EC produced a significant increase in the amount of mucus in the gastric glands. This is an efficient mechanism because the mucus barrier constitutes the first line of mucosal defense as it decreases mechanic shock, blocks bacterial access to the epithelium [33], and prevents reverse diffusion of H^+^ ions [34].

The pyloric ligation model permits the evaluation of the antisecretory actions of an experimental substance for local or systemic activity. This assay induces ulcers by an increase in gastric hydrochloric acid secretion, leading to autodigestion of the gastric mucosa and breakdown of the gastric mucosal barrier. Orally administered EC exhibited antisecretory activity by decreasing the H^+^ concentration in the gastric juice without modifying its volume, an effect which did not occur in the intraduodenally treated rats. These results lead to the conclusion that EC acts via a local rather than a systemic mechanism. Furthermore, we suggest that the decrease in H^+^ concentration in the orally treated rats can also be explained by the increase in the gastric mucus production stimulated by EC, given that the mucus barrier is able to neutralize secreted H^+^.

Gastric cells produce several antioxidants, including SOD and endogenous glutathione, which scavenge reactive oxygen species (ROS). An excessive generation of ROS enhances lipid peroxidation and depletes antioxidant enzymes [35]. SOD is one of the most effective intracellular enzymatic antioxidants, and it acts by catalyzing the dismutation of superoxide into oxygen and hydrogen peroxide [36]. Total glutathione is an important antioxidant that is essential in maintaining the integrity of the gastric mucosa. It prevents injuries caused by noxious agents through protection from free-radical-induced damage; however, its levels are reduced during the ulcerative process [37]. The results indicate that the gastroprotective effect is not related to the total glutathione pathway but to the SOD antioxidant mechanism. The antioxidant potential of EC was reinforced by previous studies showing that EC was able to prevent lipid peroxidation in the rat brain (data not shown). These important findings are evidence that the gastroprotective mechanism of EC is related not only to the reinforcement of the mucus barrier but also to an antioxidant pathway.

The increase in the immunoreactive area for NO and HSP-70 is also an important result. NO has attracted considerable attention as a gastric defensive factor. There is substantial evidence that NO, a gas signaling molecule secreted by the gastric epithelial cells, acts by increasing mucosal blood flow, regulating the secretion of mucus and bicarbonate, and inhibiting the secretion of gastric juice [38]. It is also known that HSP-70 is induced inside cells exposed to stressor agents. HSP-70 proteins refold or degrade denatured proteins produced by harmful agents [39], providing resistance to ulceration.

ATP-sensitive potassium channels (K^+^_ATP_) provide a gastric defense by inhibiting neutrophil activation and superoxide production and by enhancing gastric microcirculation [40]. Our data, however, demonstrate that K^+^_ATP_ channels are not involved in the mechanism of action of EC because the blockade of these channels with glibenclamide did not reverse the gastroprotective effects of EC.

Our investigation of gastric mucosal protective mechanisms was not focused solely on local mucosal processes. Presynaptic *α*_2_-adrenoceptors mediate several responses in the gastrointestinal tract, including antisecretory action and mucosal protective effects [41], which result in gastroprotection against different types of mucosal damage. The gastroprotection afforded by EC was reversed by yohimbine, an *α*_2_-receptor antagonist, suggesting an active role for *α*_2_-adrenoceptors in gastroprotection.

## 5. Conclusion

Taken together, these findings suggest that EC mediates gastroprotection through a mechanism that includes the reinforcement of the mucus barrier and the neutralization of gastric juices. Furthermore, this occurs with the involvement of SH compounds, presynaptic *α*_2_-adrenoceptors, SOD, NO, and HSP-70, in addition to antioxidant effects seen upon treatment with this compound.

## Figures and Tables

**Table 1:** Histological scores (0–12) and immunoreactive areas (*μ*m^2^) for HSP-70, SOD, and NO in rat stomachs (*n* = 7) with ethanol-induced gastric ulcers after treatment with vehicle, carbenoxolone (100 mg/kg), or epicatechin (50 mg/kg). The scores are represented by median (range), analyzed by ANOVA followed by Dunn, and compared to vehicle, ***P < 0.001. HSP-70, SOD, and NO are represented by the mean ± SEM, analyzed by ANOVA followed by Dunnett's test, and compared to vehicle, **P < 0.01.

Analysis	Vehicle	Carbenoxolone	Epicatechin
Score	12 (11-12)	4 (2–7)***	3 (2-3)***
HSP-70	3718.93 ± 479.00	1362.30 ± 244.50**	7861.07 ± 325.92**
SOD	15631.75 ± 956.50	12933.21 ± 581.75	26887.53 ± 971.40**
NO	10741.16 ± 302.40	11892.80 ± 324.72	13686.50 ± 581.96**

**Table 2:** Effects of epicatechin (50 mg/kg) on ethanol-induced gastric ulcer area (mm^2^) in rats (*n* = 7) pretreated with the SH reagent NEM (7 mg/kg). The results are expressed as the mean ± SEM and analyzed by an unpaired *t*-test. *P* < 0.01 compared to the control group, which was orally treated with vehicle.

Pretreatment (i.p)	Treatment (p.o)	Gastric ulcer area (mm^2^)	Percentage of gastroprotection
Vehicle	Vehicle	251.68 ± 46.59	—
	Epicatechin	30.11 ± 10.61**	88.04

NEM (7 mg/kg)	Vehicle	1157.40 ± 266.09	—
	Epicatechin	558.70 ± 86.11	51.73

**Table 3:** Effects of epicatechin (50 mg/kg) on ethanol-induced gastric ulcer area (mm^2^) in rats (*n* = 7) pretreated with glibenclamide (K^+^_ATP_ channels blocker, 3 mg/kg) or yohimbine (*α*_2_-receptor antagonist, 3 mg/kg). The results are expressed as the mean ± SEM and analyzed by an unpaired *t*-test. *P < 0.05 and **P < 0.01 compared to the control group, which was orally treated with vehicle.

Pretreatment (i.p)	Treatment (p.o)	Gastric ulcer area (mm^2^)	Percentage of gastroprotection
Vehicle	Vehicle	599.11 ± 98.78	—
	Epicatechin	243.33 ± 61.05*	59.39

Glibenclamide	Vehicle	134.13 ± 16.03	—
	Epicatechin	21.27 ± 6.00**	84.14

Yohimbine	Vehicle	602.64 ± 102.27	—
	Epicatechin	1028.81 ± 171.94	0

**Table 4:** Effects of cimetidine (100 mg/kg) or epicatechin (50 mg/kg) on gastric juice parameters in rats (*n* = 7) with pyloric ligation. The results are expressed as the mean ± SEM and analyzed by ANOVA followed by Dunnett's test. *P < 0.05 and **P < 0.01 compared to the vehicle group.

Route	Treatment	Gastric juice volume (mL)	[H^+^] mequiv/mL/4 h
Oral	Vehicle	1.22 ± 0.80	8.03 ± 1.28
	Cimetidine	1.17 ± 0.36	2.49 ± 0.47**
	Epicatechin	1.80 ± 0.55	4.56 ± 0.56*

Intraduodenal	Vehicle	3.45 ± 0.37	7.82 ± 0.45
	Cimetidine	2.08 ± 0.16**	4.20 ± 0.63**
	Epicatechin	1.82 ± 0.15**	7.64 ± 1.29

## References

[1] Laine L, Takeuchi K, Tarnawski A (2008). Gastric mucosal defense and cytoprotection: bench to bedside. *Gastroenterology*.

[2] Repetto MG, Llesuy SF (2002). Antioxidant properties of natural compounds used in popular medicine for gastric ulcers. *Brazilian Journal of Medical and Biological Research*.

[3] Chan FKL, Leung WK (2002). Peptic-ulcer disease. *The Lancet*.

[4] Blaser MJ (2008). Disappearing microbiota: *Helicobacter pylori* protection against esophageal adenocarcinoma.. *Cancer Prevention Research*.

[5] Chey WD, Wong BCY (2007). American College of Gastroenterology guideline on the management of *Helicobacter pylori* infection. *American Journal of Gastroenterology*.

[6] Fock KM, Katelaris P, Sugano K (2009). Second Asia-Pacific Consensus Guidelines for *Helicobacter pylori* infection. *Journal of Gastroenterology and Hepatology*.

[7] Malfertheiner P, Megraud F, O’Morain C (2007). Current concepts in the management of *Helicobacter pylori* infection: the Maastricht III Consensus Report. *Gut*.

[8] Paoluzi OA, Visconti E, Andrei F (2010). Ten and eight-day sequential therapy in comparison to standard triple therapy for eradicating *Helicobacter pylori* infection: a randomized controlled study on efficacy and tolerability. *Journal of Clinical Gastroenterology*.

[9] Selgrad M, Malfertheiner P (2011). Treatment of *Helicobacter pylori*. *Current Opinion in Gastroenterology*.

[10] Samie A, Obi CL, Bessong PO, Namrita L (2005). Activity profiles of fourteen selected medicinal plants from Rural Venda communities in South Africa against fifteen clinical bacterial species. *African Journal of Biotechnology*.

[11] Al-Khalil S (1995). A survey of plants used in Jordanian traditional medicine. *International Journal of Pharmacognosy*.

[12] WHO (1999). *Monographs on Selected Medicinal Plants*.

[13] Gescher A (2010). New perspectives on dietary polyphenols: taking stock, more to come. *Molecular Nutrition & Food Research*.

[14] Andreo MA, Ballesteros KVR, Hiruma-Lima CA, Machado da Rocha LR, Souza Brito ARM, Vilegas W (2006). Effect of *Mouriri pusa* extracts on experimentally induced gastric lesions in rodents: role of endogenous sulfhydryls compounds and nitric oxide in gastroprotection. *Journal of Ethnopharmacology*.

[15] Jahovic N, Erkanli G, Işeri S, Arbak S, Alican I (2007). Gastric protection by *α*-melanocyte-stimulating hormone against ethanol in rats: involvement of somatostatin. *Life Sciences*.

[16] Anderson ME (1985). Determination of glutathione and glutathione disulfide in biological samples. *Methods in Enzymology*.

[17] Puscas I, Puscas C, Coltau M (1997). Comparative study of the safety and efficacy of ebrotidine versus ranitidine and placebo in the prevention of piroxicam-induced gastroduodenal lesions. *Arzneimittel-Forschung/Drug Research*.

[18] Shay H, Komarov SA, Fels SS, Meranze D, Gruenstein M, Siplet H (1945). A simple method for the uniform production of gastric ulceration in the rat. *Gastroenterology*.

[19] Rafatullah S, Tariq M, Al-Yahya MA, Mossa JS, Ageel AM (1990). Evaluation of turmeric (*Curcuma longa*) for gastric and duodenal antiulcer activity in rats. *Journal of Ethnopharmacology*.

[20] Motawi TK, Abd Elgawad HM, Shahin NN (2008). Gastroprotective effect of leptin in indomethacin-induced gastric injury. *Journal of Biomedical Science*.

[21] Yoshikawa T, Naito Y, Kishi A (1993). Role of active oxygen, lipid peroxidation, and antioxidants in the pathogenesis of gastric mucosal injury induced by indomethacin in rats. *Gut*.

[22] Fermin Sanchez De Medina LH, Gálvez J, Romero JA, Zarzuelo A (1996). Effect of Quercitrin on acute and chronic experimental colitis in the rat. *Journal of Pharmacology and Experimental Therapeutics*.

[23] Crespo ME, Gálvez J, Cruz T, Ocete MA, Zarzuelo A (1999). Anti-inflammatory activity of diosmin and hesperidin in rat colitis induced by TNBS. *Planta Medica*.

[24] García-Argáez AN, Ramírez Apan TO, Delgado HP, Velázquez G, Martínez-Vázquez M (2000). Anti-inflammatory activity of coumarins from Decatropis bicolor on TPA ear mice model. *Planta Medica*.

[25] Cruz T, Gálvez J, Crespo E, Ocete MA, Zarzuelo A (2001). Effects of Silymarin on the acute stage of the trinitrobenzenesulphonic acid model of rat colitis. *Planta Medica*.

[26] Di Stasi LC, Camuesco D, Nieto A, Vilegas W, Zarzuelo A, Galvez J (2004). Intestinal anti-inflammatory activity of paepalantine, an isocoumarin isolated from the capitula of Paepalanthus bromelioides, in the trinitrobenzenesulphonic acid model of rat colitis. *Planta Medica*.

[27] Siegmund S, Haas S, Schneider A, Singer MV (2003). Animal models in gastrointestinal alcohol research—a short appraisal of the different models and their results. *Bailliere’s Best Practice and Research in Clinical Gastroenterology*.

[28] Bafna PA, Balaraman R (2004). Anti-ulcer and antioxidant activity of DHC-1, a herbal formulation. *Journal of Ethnopharmacology*.

[29] Al-Howiriny T, Alsheikh A, Alqasoumi S, Al-Yahya M, ElTahir K, Rafatullah S (2009). Protective effect of *Origanum majorana* L. “Marjoram” on various models of gastric mucosal injury in rats. *American Journal of Chinese Medicine*.

[30] Szabo S, Vattay P (1990). Experimental gastric and duodenal ulcers: advances in Pathogenesis. *Gastroenterology Clinics of North America*.

[31] Salim AS (1993). Sulfhydryl-containing agents: new approach to the problem of refractory peptic ulceration. *Pharmacology*.

[32] Avila JR, Alarcón De La Lastra C, Martín MJ (1996). Role of endogenous sulphydryls and neutrophil infiltration in the pathogenesis of gastric mucosal injury induced by piroxicam in rats. *Inflammation Research*.

[33] Belley A, Keller K, Göettke M, Chadee K (1999). Intestinal mucins in colonization and host defense against pathogens. *The American Journal of Tropical Medicine and Hygiene*.

[34] Banerjee D, Hassarajani SA, Maity B, Narayan G, Bandyopadhyay SK, Chattopadhyay S (2010). Comparative healing property of kombucha tea and black tea against indomethacin-induced gastric ulceration in mice: possible mechanism of action. *Food and Function*.

[35] Cadirci E, Suleyman H, Aksoy H (2007). Effects of *Onosma armeniacum* root extract on ethanol-induced oxidative stress in stomach tissue of rats. *Chemico-Biological Interactions*.

[36] Aviello G, Canadanovic-Brunet JM, Milic N (2011). Potent antioxidant and genoprotective effects of boeravinone G, a rotenoid isolated from *Boerhaavia diffusa*. *PLoS One*.

[37] Li CY, Xu HD, Zhao BT, Chang HI, Rhee HI (2008). Gastroprotective effect of cyanidin 3-glucoside on ethanol-induced gastric lesions in rats. *Alcohol*.

[38] Petersson J, Phillipson M, Jansson EÅ, Patzak A, Lundberg JO, Holm L (2007). Dietary nitrate increases gastric mucosal blood flow and mucosal defense. *American Journal of Physiology*.

[39] Morimoto RI, Gabriella Santoro M (1998). Stress-inducible responses and heat shock proteins: new pharmacologic targets for cytoprotection. *Nature Biotechnology*.

[40] Campos DA, De Lima AF, Ribeiro SRL (2008). Gastroprotective effect of a flavone from *Lonchocarpus araripensis* Benth. (Leguminosae) and the possible mechanism. *Journal of Pharmacy and Pharmacology*.

[41] Gyires K, Müllner K, Fürst S, Rónai AZ (2000). Alpha-2 adrenergic and opioid receptor-mediated gastroprotection. *Journal of Physiology Paris*.

